# Evaluating risk factors associated with COVID-19 infections among vaccinated people early in the U.S. vaccination campaign: an observational study of five states, January–March 2021

**DOI:** 10.1186/s12879-022-07702-x

**Published:** 2022-09-01

**Authors:** Katrin S. Sadigh, Kiersten J. Kugeler, Sara Bressler, Stephanie C. Massay, Emma Schmoll, Lauren Milroy, Alyson M. Cavanaugh, Allison Sierocki, COVID-19 Vaccine Breakthrough Characterization Team, Layne Dorough, Kiren Mitruka, Kristin Lecy, Rebekah Porter, Louisa Castrodale, Wendy M. Bamberg, Nisha Alden, Andzelika Rzucidlo, Kevin B. Spicer, Taylor Miller, Augustus E. Madsen, Claire Holladay, Benjamin D. Scott, Cassandra Jones, Brittany Eziam, Jacqueline Logan, Caleb Wiedeman, Marc Fischer, Leisha D. Nolen

**Affiliations:** 1 Centers for Disease Control and Prevention, 1600 Clifton Rd, Atlanta, GA 30329 USA; 2 Epidemic Intelligence Service, Center for Disease Control and Prevention, 1600 Clifton Rd, Atlanta, GA 30329 USA; 3 Centers for Disease Control and Prevention, 3156 Rampart Rd, Fort Collins, CO 80521 USA; 4 Centers for Disease Control and Prevention, 4055 Tudor Centre Dr, Anchorage, AK 99508 USA; 5 Alaska Department of Health and Social Services, 3601 C St, Anchorage, AK 99503 USA; 6 Colorado Department of Public Health and Environment, 4300 Cherry Creek South Dr, Denver, CO 80246 USA; 7 Indiana Department of Health, 2 N. Meridian St, Indianapolis, IN 46204 USA; 8 Kentucky Department for Public Health, 275 E Main St, Frankfort, KY 40601 USA; 9 Tennessee Department of Health, 710 James Robertson Parkway, Nashville, TN 37243 USA

**Keywords:** SARS-CoV-2, COVID-19 vaccines, Vaccine breakthrough infections, Vaccination

## Abstract

**Background:**

COVID-19 vaccines are an effective tool to prevent illness due to SARS-CoV-2 infection. However, infection after vaccination still occurs. We evaluated all infections identified among recipients of either the Pfizer-BioNTech or Moderna COVID-19 vaccine in five U.S. states during January–March 2021.

**Methods:**

Using observational data reported to CDC, we compared the incidence of SARS-CoV-2 infection among vaccinated and unvaccinated persons, and the sex, age, and vaccine product received for individuals with vaccine breakthrough infections to those of the vaccinated population using Poisson regression models. We also compared the proportion of vaccine breakthrough cases due to a SARS-CoV-2 variant of concern to data reported to CDC’s national genomic surveillance program.

**Results:**

The age-adjusted incidence of reported SARS-CoV-2 infection was 97% lower among vaccinated as compared to unvaccinated persons aged ≥ 16 years (68 vs 2252 cases per 100,000 people). Vaccinated adults aged ≥ 85 years were 1.6 times (95% CI 1.3–1.9) as likely to become infected with SARS-CoV-2 than vaccinated adults aged < 65 years. Pfizer-BioNTech COVID-19 vaccine recipients were 1.4 times (95% CI 1.3–1.6) as likely to experience infection compared to Moderna COVID-19 recipients. The proportion of infections among vaccinated persons caused by SARS-CoV-2 variants of concern was similar to the proportion of circulating viruses identified as variants of concern in the five states during the same time.

**Conclusions:**

Vaccinated persons had a substantially lower incidence of SARS-CoV-2 infection compared to unvaccinated persons. Adults aged ≥ 85 years and Pfizer-BioNTech vaccine recipients had a higher risk of infection following vaccination. We provide an analytic framework for ongoing evaluation of patterns associated with SARS-CoV-2 infection among vaccinated persons using observational surveillance and immunization data. Our findings reinforce the effectiveness of COVID-19 vaccines in preventing infection in real-world settings.

**Supplementary Information:**

The online version contains supplementary material available at 10.1186/s12879-022-07702-x.

## Background

COVID-19 vaccines are a critical tool for controlling the global pandemic [1]. The U.S. Food and Drug Administration (FDA) has approved or issued Emergency Use Authorizations for multiple COVID-19 vaccines [2]. In large, randomized controlled trials, vaccine efficacy was ≥ 94% for Pfizer-BioNTech and Moderna COVID-19 vaccines and 66% for Johnson & Johnson’s Janssen COVID-19 vaccine [3–6]. Additional studies have confirmed the effectiveness of these vaccines in real-world settings [7–12]. Despite high vaccine effectiveness, some fully vaccinated people will develop asymptomatic SARS-CoV-2 infection or symptomatic COVID-19, also referred to as “vaccine breakthrough” infections [3–5, 7–13]. In addition, emergence of new SARS-CoV-2 variants could impact protection provided by vaccines [14, 15].

A previous report summarized the first ~ 10,000 COVID-19 vaccine breakthrough infections reported to Centers for Disease Control and Prevention (CDC) from 46 U.S. states and territories from January to April 2021 [13]. However, the national surveillance system relies on voluntary and passive reporting and data might not be complete or representative. In addition, the report did not include denominator data on vaccinated individuals, information that provides crucial context for interpretation of the findings.

To better understand risk factors associated with COVID-19 infections among vaccinated persons, we evaluated cases that occurred during January–March 2021 in five states in the context of the vaccinated population within the same states during the first few months of the U.S. vaccination campaign. We also compared SARS-CoV-2 sequence data for the reported COVID-19 infections among vaccinated persons to specimens submitted from the five states to CDC’s national genomic surveillance program.

## Methods

### Definitions and inclusion criteria

Vaccinated persons were those with ≥ 14 days after completion of the recommended primary series of an FDA-authorized COVID-19 vaccine. At the time of study, only Pfizer-BioNTech and Moderna COVID-19 vaccines were FDA-authorized, both of which had a two-dose primary series. Unvaccinated persons were those who did not meet criteria above, and thus included persons who were not vaccinated or were partially vaccinated with the primary series, including those who had received the primary series but were < 14 days following series completion at time of analysis.

A COVID-19 case in a vaccinated person was defined as the detection of SARS-CoV-2 RNA or antigen in a respiratory specimen during the study time period. Analysis included cases who completed their primary vaccine series by February 28 and tested positive for SARS-CoV-2 by March 31, 2021.

### COVID-19 case identification among the vaccinated population and data collection

Five states (Alaska, Colorado, Indiana, Kentucky, and Tennessee) were selected because of their similar approach to case identification through active linkage of their COVID-19 case surveillance systems with their immunization information systems, which was relatively rare among states at the time of this analysis. State health department personnel actively identified COVID-19 cases among vaccinated individuals by matching all positive SARS-CoV-2 laboratory test results with state-based immunization information systems using patient name and date of birth. De-identified cases among vaccinated people were reported to CDC’s COVID-19 vaccine breakthrough surveillance database; data on cases used in this analysis were subjected to additional scrutiny for completeness and illness outcome by participating health departments and sent to CDC by June 2021 [16, 17]. Variables included patient demographics, residence location and type, SARS-CoV-2 laboratory test method, COVID-19 vaccination type and dates, hospitalization, and outcome. State-based hospitalization and death databases were reviewed to ascertain severe outcomes. For fatal cases, hospital records and death certificates were reviewed to categorize deaths as COVID-19 related or non-related. Available respiratory specimens that tested positive for SARS-CoV-2 RNA were characterized by viral genomic analysis [18]. Vaccine administration data for vaccinated individuals by week were obtained from immunization information systems of each state.

This activity was reviewed at CDC, determined to be non-research public health investigation not requiring further institutional review board review and was conducted in accordance with applicable federal law and CDC policy.

### Data analysis

Data were aggregated across the five participating states and analyzed at CDC using SAS version 9.4 (SAS Institute, Cary, NC). Patient age was categorized into five groups for descriptive analyses: 16–49, 50–64, 65–74, 75–84, and ≥ 85 years. Race and ethnicity data were combined. Where residential status was known, nursing homes and assisted living facilities were categorized as long-term care facilities.

Person-weeks at risk for COVID-19 among the vaccinated population was calculated by multiplying the count of vaccinated persons in each stratum of week of vaccine completion, sex, age group, and vaccine type by the total number of weeks at risk beginning 14 days after receipt of the second vaccine dose through the end of March 2021.

We compared the rate of COVID-19 cases among the vaccinated population in the five states during January–March 2021 to the rate of reported COVID-19 cases among the unvaccinated population ≥ 16 years of age. The number of COVID-19 cases that occurred among the unvaccinated population was not directly available and was thus approximated by subtracting the number of COVID-19 cases among vaccinated persons and vaccinated population counts from the five states from their total COVID-19 case counts during January–March 2021 and the total estimated 2019 state population, respectively. Rates of COVID-19 among vaccinated and unvaccinated populations were directly standardized to the 2010 U.S. population in four age categories: 16–49, 50–64, 65–74, and 75 years and older [19, 20].

To evaluate if COVID-19 among vaccinated persons was more likely to occur among certain populations, we compared the sex, age, and vaccine product received for COVID-19 cases to those of the vaccinated population within each state. For this analysis, we further collapsed age groups into three categories: 16–64, 65–84, and ≥ 85 years. We calculated incidence rates of infection among vaccinated persons according to person-weeks at risk in each stratum and used Poisson models to calculate incidence rate ratios (IRR) and 95% confidence intervals (CI). We used a multivariable Poisson model to generate IRRs adjusted for each variable of interest as well as for potential confounding by state of residence and month of vaccine series completion to account for differences in SARS-CoV-2 transmission across states and over time. Multivariable analyses did not include race, ethnicity, residential housing status or occupation, as incomplete or unavailable data in these categories precluded reliable IRR analysis. Sensitivity analyses with removal of epidemiologically linked cases in the same long-term care facility and those reported as asymptomatic were performed to evaluate their respective impact on overall findings. Infections among vaccinated persons were not removed from the denominator of vaccinated persons eligible for vaccine breakthrough in weeks following occurrence of their infection given their small number compared to the total vaccinated population. Due to sample size, only ratios of infection rates according to vaccination status were compared, not rates of severe outcomes.

### Sequence data analysis

SARS-CoV-2 sequence data from the representative sample of specimens routinely submitted to CDC’s national genomic surveillance program from the five states were used to characterize viral strains circulating in those areas during the study period [21, 22]. We used the chi-square test to compare the infections among fully vaccinated persons due to a SARS-CoV-2 variant of concern to the proportions of variants of concern in the genomic surveillance data [14]. A sensitivity analysis was performed that matched each SARS-CoV-2 case with sequence data to one randomly selected case from the same state and month with sequence data reported to the national genomic surveillance program. A second sensitivity analysis was performed that excluded viral sequence data from epidemiologically linked cases.

## Results

As of March 31, 2021, the age-adjusted rate of SARS-CoV-2 infections among vaccinated individuals was 68 cases per 100,000 persons in the five states. In contrast, among an estimated 22,429,778 people in the five states who were unvaccinated, a total of 676,087 cases occurred during January–March 2021. After restricting to the vaccine-eligible population (aged ≥ 16 years), the age-adjusted rate of SARS-CoV-2 infection among people who were not fully vaccinated individuals was 2252 cases per 100,000 persons in the five states. This corresponds to an IRR of 33, and a 97% lower rate of infection among vaccinated persons than among not fully vaccinated persons.

Among the 1582 SARS-CoV-2 infections in vaccinated persons reported by the end of March 2021, 1053 (67%) were among females and the median age was 57 years (interquartile range, 40–75 years) (Table 1). Most cases (65%) were among White, non-Hispanic persons; however, race and/or ethnicity data were missing for 297 (19%) of the reported cases. At least 227 (14%) infections were among residents of a long-term care facility. Receipt of Pfizer-BioNTech vaccine was reported for 1073 (68%) and Moderna vaccine in 502 (32%) cases. Of the 1582 SARS-CoV-2 infections among vaccinated persons, 531 (34%) were reported as asymptomatic, 181 (11%) patients were known to be hospitalized, and 42 (3%) died. Among the 227 infections among residents of long-term care facilities, 117 (52%) were reported to be asymptomatic. Among the 181 hospitalized patients, 65 (36%) were reported as asymptomatic and 79 (44%) were hospitalized for a reason unrelated to COVID-19. The median age of patients who died was 80 years (interquartile range, 73–87 years); five (12%) decedents were asymptomatic and nine (21%) died from a cause unrelated to COVID-19. Overall, 95 (6%) of the SARS-CoV-2 infections among vaccinated persons were part of 27 clusters of epidemiologically linked cases in long-term care facilities. A median of 2.5 cases (range 2–22 cases) were identified in each cluster.

**Table 1:** COVID-19 vaccine breakthrough cases reported to CDC from five U.S. states during January–March 2021

Characteristic	Vaccine breakthrough cases [N = 1582]	
	No	(%)
Gender		
Female	1053	(67)
Male	524	(33)
Unknown	5	(< 1)
Age group in years		
16–49	632	(40)
50–64	333	(21)
65–74	226	(14)
75–84	235	(15)
≥ 85	156	(10)
Race/ethnicity		
Non-Hispanic		
White	1024	(65)
Black	60	(4)
American Indian/Alaska Native	52	(3)
Asian	24	(2)
Multiracial/other	50	(3)
Hispanic, any race	75	(5)
Unknown	297	(19)
Residence type		
Long-term care facility	227	(14)
Other	956	(60)
Unknown	399	(25)
Positive SARS-CoV-2 test type		
Nucleic acid amplification test	1312	(83)
Antigen	254	(16)
Unknown	16	(1)
Vaccine received		
Pfizer-BioNTech	1073	(68)
Moderna	502	(32)
Unknown	7	(< 1)
Symptoms associated with positive test		
Asymptomatic	531	(34)
Symptomatic	661	(41)
Unknown	390	(25)
Hospitalized		
Yes^†^	181	(11)
Outcome		
Died^‡^	42	(3)

Five states are Alaska, Colorado, Indiana, Kentucky, and Tennessee

^†^Hospitalization status was reported as unknown for 387 vaccine breakthrough cases but they were not identified in the hospitalization databases

^‡^Outcome was reported as unknown for 450 vaccine breakthrough cases but they were not identified in the death databases

Overall, vaccinated people were more frequently female, < 65 years of age, and received the Pfizer-BioNTech vaccine (Table 2). The incidence rate among vaccinated females was 14.6 per 100,000 person-weeks compared to 12.3 per 100,000 among males; however, after adjusting for age, vaccine product, state, and month, the difference was not statistically significant (adjusted IRR 1.1, 95% CI 1.0–1.3) (Table 2). Vaccinated adults aged ≥ 85 years had a rate of 17.9 infections per 100,000 person-weeks with an adjusted IRR of 1.6 (95% CI 1.3–1.9) compared to vaccinated adults aged < 65 years. After adjusting for other factors, the rate of infection was 1.4 times higher (95% CI 1.3–1.6) among recipients of the Pfizer-BioNTech vaccine compared to the Moderna vaccine.

**Table 2:** Sex, age group, and vaccine type for reported SARS-CoV-2 infections among vaccinated persons compared to the vaccinated population for five U.S. states during January–March 2021

	Vaccine breakthrough cases	Total vaccinated	Person-weeks of vaccinated time at risk	Rate*	Unadjusted incidence rate ratio		Adjusted incidence rate ratio**	
	No	No			IRR	(95% CI)	aIRR	(95% CI)
Sex								
Male	524	794,033	4,243,156	12.3	Ref		Ref	
Female	1,053	1,286,750	7,198,863	14.6	1.2	(1.0–1.4)	1.1	(1.0–1.3)
Age group								
< 65 years	965	1,001,871	6,387,727	15.1	Ref		Ref	
65–84 years	461	908,446	4,236,965	10.9	0.7	(0.6–0.8)	0.9	(0.8–1.1)
≥ 85 years	156	179,215	869,710	17.9	1.2	(0.9–1.5)	1.6	(1.3–2.0)
Vaccine type								
Moderna	502	951,087	4,805,959	10.4	Ref		Ref	
Pfizer	1,073	1,136,729	6,678,125	16.1	1.5	(1.3–1.8)	1.4	(1.2–1.6)

IRR: incidence rate ratio; aIRR: adjusted incidence rate ratio; CI: confidence intervals. Unknown values in numerator and denominator excluded from analyses

^*^Per 100,000 person-weeks

^**^Adjusted model included sex, age group, vaccine type, state, and month of vaccine series completion

Adjusted IRR point estimates and CIs did not substantially change when multivariable analysis was performed eliminating epidemiologically linked cases or those reported as asymptomatic (Additional file 1). State-specific multivariable models revealed similar patterns across sex and age. However, the adjusted ratio of infections among vaccinated persons associated with Pfizer-BioNTech vaccine relative to Moderna vaccine was below 1.0 in two states, slightly above 1.0 in one state, and ≥ 2.0 in two states (Additional file 2).

Sequence data were available from 155 (10%) of the 1582 infections among vaccinated persons (Table 3). Of those, 43 (28%) were due to a SARS-CoV-2 variant of concern, including B.1.1.7 (Alpha) (41; 26%) and P.1 (Gamma) (2; 1%). There were no statistically significant differences between the proportion of infections among vaccinated persons due to a variant of concern and the proportion of sequences that were variants of concern reported to the national genomic surveillance program from the five states during the same time. These findings did not change in a matched analysis from the same state and month, or after excluding linked cases.

**Table 3:** SARS-CoV-2 sequence results for reported vaccine breakthrough cases and national genomic surveillance program from five states during January–March 2021

SARS-CoV-2 lineages	Vaccine breakthrough case [N = 155]		Genomic surveillance data [N = 5738]	
	No	(%)	No	(%)
Variants of concern				
B.1.1.7 (Alpha)	41	(26)	1,649	(29)
P.1 (Gamma)	2	(1)	29	(1)
B.1.351 (Beta)	0	(0)	11	(< 1)
B.1.617.2 (Delta)	0	(0)	1	(< 1)
Other lineages	112	(72)	4,048	(71)

Chi-square p-value > 0.05 for differences between the SARS-CoV-2 sequence data for reported vaccine breakthrough cases and sequence data reported from the five states to the national genomic surveillance program

## Discussion

In five states early in the US vaccination campaign, risk of SARS-CoV-2 infection among vaccinated residents occurred at a rate 97% lower than that among unvaccinated or partially vaccinated people. Reported cases of SARS-CoV-2 among vaccinated persons were more commonly female, White, non-Hispanic persons, generally reflecting the population vaccinated during this timeframe; however, these cases were more likely to occur among persons ≥ 85 years of age. The proportion of infections among vaccinated persons caused by SARS-CoV-2 variants of concern was similar to the proportion of these variants circulating in these states in the first 3 months of 2021.

An elevated rate of SARS-CoV-2 infection among vaccinated persons was identified among those who received the Pfizer-BioNTech vaccine compared to those who received the Moderna vaccine. However, this finding may be an artifact due to vaccine distribution and routine testing in certain populations that could not be accounted for analytically. Specifically, Pfizer-BioNTech vaccine was selected by three of the five states for mass distribution in long-term care facilities at the beginning of the U.S. COVID-19 vaccination roll-out. In state-specific analyses, an elevated rate of infection among fully vaccinated persons who were Pfizer-BioNTech vaccine recipients was detected only in the three states that utilized that vaccine for their long-term care facility mass vaccination roll-out, suggesting the elevated IRR calculated for the Pfizer-BioNTech vaccine may be a product of which vaccine was utilized for vaccination in long-term care facilities in the specific states under study. While most data from vaccine effectiveness studies support clinical trial data showing high levels of protection for both vaccines, recent research has demonstrated higher vaccine effectiveness over time for Moderna compared to Pfizer-BioNTech vaccine [7–12, 23–26]. Other characteristics of initial COVID-19 infections among vaccinated persons likely were influenced by the targeting of healthcare workers for early vaccine roll-out. Healthcare workers are at higher risk for SARS-CoV-2 exposure, undergo more frequent routine testing, and are > 80% female, possibly skewing the frequency of reported SARS-CoV-2 infections among females [7]. With ongoing evaluation and analysis, the influence of residual confounding among select subgroups on overall patterns may be minimized.

A higher rate of SARS-CoV-2 infections after vaccination among the oldest adults compared to younger persons is not unexpected and is seen with other vaccines, such as the influenza vaccine [27]. Despite a higher rate of vaccine breakthrough infections compared to younger adults, data have shown that COVID-19 vaccination is highly effective at preventing morbidity and mortality among the oldest adults, highlighting the importance of ensuring high vaccine uptake in this population despite elevated risk for infection following vaccination [8, 23, 28].

We found no evidence of increased likelihood of infections among vaccinated persons associated with specific SARS-CoV-2 variants of concern, although genomic sequence data were only available for 10% of reported infections among fully vaccinated persons, limiting our ability to draw conclusions from these patterns. Importantly, this early evaluation was conducted prior to the emergence of the B.1.617.2 (Delta) or B.1.1.529 (Omicron) variants, which were linked to reduced vaccine effectiveness against SARS-CoV-2 infection while maintaining high but waning effectiveness against severe outcomes [29–32]. Observational surveillance data is prone to bias, but the sheer sample size captured provides valuable information on patterns of COVID-19 according to vaccination status over time, particularly among older adults. CDC is partnering with health departments to actively link their case surveillance and immunization registry data to monitor patterns of COVID-19 according to vaccination status over time. Ongoing analysis of surveillance data complements smaller, controlled studies that measure vaccine effectiveness [29–32]. The incidence of expected infections among vaccinated persons will vary with COVID-19 vaccine coverage and disease incidence and will increase if circulation of specific variants reduces the relative protection afforded by vaccines.

### Limitations

The findings of this analysis are subject to several limitations. We sought to control for confounding although residual confounding in these observational data is expected. Healthcare workers and residents of long-term care facilities likely remain overrepresented in this analysis, as they were among the first people to be fully vaccinated in the United States. Data were incomplete for many variables, including race and ethnicity, presence of COVID-19 symptoms, hospitalization, and illness outcome, limiting our ability to draw conclusions regarding the distribution of these variables among vaccine breakthrough cases. States used a similar approach to case identification by system linkage that minimized but did not eliminate potential for under ascertainment of vaccine history among COVID-19 cases. Risk factors for SARS-CoV-2 infection among vaccinated persons in these states may not be generalizable across the United States. Our calculated rate of COVID-19 among the unvaccinated population in these five states during this time frame is an approximation and an unknown proportion of partially vaccinated persons would inherently be included in both the numerator and denominator of that rate, possibly yielding an underestimate of the rate of COVID-19 in the unvaccinated population. Lastly, despite active linkage of surveillance and immunization information systems, vaccinated individuals with mild symptoms or who were asymptomatic may have been less likely to seek SARS-CoV-2 testing; home-based SARS-CoV-2 testing was not widely available during the study period, though this is unlikely to have contributed to under ascertainment of SARS-CoV-2 to any substantial degree. In contrast, severe cases were more likely to be detected and reported, so proportions of infections among vaccinated persons associated with severe outcomes are likely an overestimate.

## Conclusions

Despite the nearly 220 million U.S. residents who have been vaccinated by April 2022, SARS-CoV-2 transmission persists. SARS-CoV-2 infections among vaccinated persons are expected to occur but the risk of infection and severe disease is substantially higher among unvaccinated persons [6–12]. Our analysis affirms that vaccines prevent SARS-CoV-2 infection across all demographic groups though adults ≥ 85 years had a higher risk of infection following vaccination. We provide an analytic framework for ongoing evaluation of patterns associated with SARS-CoV-2 infection among vaccinated persons using observational surveillance and immunization data.

## Supplementary Information

**Additional file 1.** Sensitivity analyses for adjusted incidence rate ratios for sex, age group, and vaccine type for reported SARS-CoV-2 infection among fully vaccinated persons among five U.S. states during January–March 2021.

**Additional file 2.** COVID-19 vaccine breakthrough case data and vaccinated population data used for analyses, stratified by state, vaccine manufacturer, age category, sex, week of second vaccine dose, case count, and person-weeks at risk.

## Data Availability

The authors declare that the data supporting the findings of this study are available within the article and its supplementary information files.

## Abbreviations

COVID-19: Coronavirus disease 2019
U.S.: United States
FDA: Food and Drug Administration
SARS-CoV-2: Severe acute respiratory syndrome coronavirus-2
CDC: Centers for Disease Control and Prevention
CI: Confidence intervals
IRR: Incidence rate ratios
aIRR: Adjusted incidence rate ratio
