# Supplementary material for: Evaluating risk factors associated with COVID-19 infections among vaccinated people early in the U.S. vaccination campaign: an observational study of five states, January–March 2021
Source: BMC Infect Dis. 2022 Sep 1;22:718. doi: 10.1186/s12879-022-07702-x (PMC9434543; doi:10.1186/s12879-022-07702-x)
Supplement: Supplementary file 1 — Additional file 1. Sensitivity analyses for adjusted incidence rate ratios for sex, age group, and vaccine type for reported SARS-CoV-2 infection among fully vaccinated persons among five U.S. states during January–March 2021. [file 12879_2022_7702_MOESM1_ESM.docx]

**Supplementary material.** Sensitivity analyses for adjusted incidence rate ratios for sex, age group, and vaccine type for reported SARS-CoV-2 infection among fully vaccinated persons among five U.S. states during January–March 2021.

**Supplementary Table 1.** **Adjusted* incidence rate ratios from primary model and when removing breakthrough cases associated with clusters and removing asymptomatic cases from the analysis**

|  | **Primary adjusted model (as in Table 2)** | | | **Adjusted model removing cases associated with clusters** | | **Adjusted model removing asymptomatic cases** | |
| --- | --- | --- | --- | --- | --- | --- | --- |
|  | IRR | (95% CI) | | IRR | (95% CI) | IRR | (95% CI) |
| **Sex** |  | | |  | |  | |
| Male | Ref |  | | Ref |  | Ref |  |
| Female | 1.1 | (1.0–1.3) | | 1.1 | (1.0–1.3) | 1.3 | (1.1–1.5) |
| **Age** |  | | |  | |  | |
| <65 years | Ref |  |  | Ref |  | Ref |  |
| 65-84 years | 0.9 | (0.8–1.1) | | 0.9 | (0.7–1.0) | 0.8 | (0.7–1.0) |
| ≥85 years | 1.6 | (1.3–2.0) | | 1.4 | (1.1–1.8) | 1.5 | (1.1–1.9) |
| **Vaccine type** |  | | |  | |  | |
| Moderna | Ref |  | | Ref |  | Ref |  |
| Pfizer | 1.4 | (1.2–1.6) | | 1.4 | (1.2–1.6) | 1.5 | (1.3–1.8) |

*Adjusted model included sex, age group, vaccine type, state, and month of vaccine series completion

**Supplementary Table 2. Adjusted* incidence rate ratios for state-specific models**

|  | | Adjusted model State 1 only | | Adjusted model State 2 only | | | Adjusted model State 3 only | | | Adjusted model State 4 only | | | Adjusted model state 5 only | |
| --- | --- | --- | --- | --- | --- | --- | --- | --- | --- | --- | --- | --- | --- | --- |
|  | | IRR | (95% CI) | | IRR | (95% CI) | IRR | | (95% CI) | IRR | (95% CI) | | IRR | (95% CI) |
| Sex | | | | | | | | | | | | | | |
| Male | Ref | |  | Ref | |  | Ref |  | | Ref | |  | Ref |  |
| Female | 1.1 | | (0.8–1.5) | 1.2 | | (0.9–1.5) | 1.1 | (0.9–1.5) | | 1.0 | | (0.7–1.2) | 1.2 | (1.0–1.5) |
| Age group | | | | | | | | | | | | | | |
| <65 years | Ref | |  | Ref | |  | Ref |  | | Ref | |  | Ref |  |
| 65-84 years | 0.9 | | (0.6–1.3) | 0.6 | | (0.4–0.8) | 0.8 | (0.6–1.1) | | 1.7 | | (1.3–2.4) | 0.7 | (0.6–0.9) |
| ≥85 years | 1.3 | | (0.6–2.8) | 1.1 | | (0.6–1.9) | 1.6 | (1.0–2.4) | | 2.0 | | (1.4–3.0) | 0.9 | (0.7–1.3) |
| Vaccine type | | | | | | | | | | | | | | |
| Moderna | Ref | |  | Ref | |  | Ref | |  | Ref |  | | Ref |  |
| Pfizer | 1.4 | | (0.9–2.1) | 2.0 | | (1.6–2.5) | 0.9 | | (0.7–1.1) | 2.2 | (1.7–3.0) | | 0.9 | (0.7–1.1) |

*Adjusted state-specific model included sex, age group, vaccine type, and month of vaccine series completion
